# Patient-specific identification of genome-wide DNA-methylation differences between intracranial and extracranial melanoma metastases

**DOI:** 10.1038/s41598-022-24940-w

**Published:** 2023-01-09

**Authors:** Theresa Kraft, Konrad Grützmann, Matthias Meinhardt, Friedegund Meier, Dana Westphal, Michael Seifert

**Affiliations:** 1 Institute for Medical Informatics and Biometry (IMB), Carl Gustav Carus Faculty of Medicine, Technische Universität Dresden, 01307 Dresden, Germany; 2 Department of Pathology, Carl Gustav Carus Faculty of Medicine, Technische Universität Dresden, 01307 Dresden, Germany; 3 Department of Dermatology, Carl Gustav Carus Faculty of Medicine, Technische Universität Dresden, 01307 Dresden, Germany; 4 National Center for Tumor Diseases (NCT), D-01307 Dresden, Germany

**Keywords:** Cancer genomics, Metastasis, Skin cancer, Cancer, Computational biology and bioinformatics, Data mining, Microarrays

## Abstract

Melanomas frequently metastasize to distant organs and especially intracranial metastases still represent a major clinical challenge. Epigenetic reprogramming of intracranial metastases is thought to be involved in therapy failure, but so far only little is known about patient-specific DNA-methylation differences between intra- and extracranial melanoma metastases. Hierarchical clustering of the methylomes of 24 patient-matched intra- and extracranial melanoma metastases pairs revealed that intra- and extracranial metastases of individual patients were more similar to each other than to metastases in the same tissue from other patients. Therefore, a personalized analysis of each metastases pair was done by a Hidden Markov Model to classify methylation levels of individual CpGs as decreased, unchanged or increased in the intra- compared to the extracranial metastasis. The predicted DNA-methylation alterations were highly patient-specific differing in the number and methylation states of altered CpGs. Nevertheless, four important general observations were made: (i) intracranial metastases of most patients mainly showed a reduction of DNA-methylation, (ii) cytokine signaling was most frequently affected by differential methylation in individual metastases pairs, but also MAPK, PI3K/Akt and ECM signaling were often altered, (iii) frequently affected genes were mainly involved in signaling, growth, adhesion or apoptosis, and (iv) an enrichment of functional terms related to channel and transporter activities supports previous findings for a brain-like phenotype. In addition, the derived set of 17 signaling pathway genes that distinguished intra- from extracranial metastases in more than 50% of patients included well-known oncogenes (e.g. *PRKCA*, *DUSP6*, *BMP4*) and several other genes known from neuronal disorders (e.g. *EIF4B*, *SGK1*, *CACNG8*). Moreover, associations of gene body methylation alterations with corresponding gene expression changes revealed that especially the three signaling pathway genes *JAK3*, *MECOM*, and *TNXB* differ strongly in their expression between patient-matched intra- and extracranial metastases. Our analysis contributes to an in-depth characterization of DNA-methylation differences between patient-matched intra- and extracranial melanoma metastases and may provide a basis for future experimental studies to identify targets for new therapeutic approaches.

## Introduction

Melanomas represent the most aggressive form of skin cancer and their occurrence has increased over the past few years^1–3^. Melanoma development is triggered by DNA mutations in melanocytes, which are mainly induced by solar ultraviolet radiation^4^. Especially *BRAF* mutations are frequently involved in the development of melanomas by enhancing cell proliferation through an uncontrolled activation of the MAPK signaling pathway^5–7^. Melanomas also frequently metastasize to other organs like lymph node, lung, liver or brain^3,8^. In fact, intracranial metastases are observed in about 50% of metastatic melanoma patients^9^ and without treatment affected patients only survive on average about four months^10^.

There are several treatment options for metastatic melanoma including BRAF and MEK inhibitors or immune checkpoint inhibitors^11,12^. These approved therapies significantly improve the survival of patients with extracranial metastases^13–15^. However, for intracranial metastases, the response duration of BRAF/MEK inhibitors is limited to a few months and the efficacy of immune checkpoint inhibitors is substantially reduced in symptomatic patients^9,16–20^. Thus, intracranial metastases are the leading cause of death for melanoma patients^21^. Therefore, more detailed knowledge about molecular differences between intra- and extracranial metastases is required to provide a better basis for the development of therapeutic strategies for intracranial metastases.

Over the last years, several studies were done to better understand differences between intra- and extracranial metastases at the molecular level. Three studies identified an upregulation of the PI3K/Akt pathway in intra- compared to extracranial metastases^22–24^. Several studies analyzed the gene mutation landscape with different results. Recently, genes (e.g. *ARID1A*, *ARID2*, *SMARCA4*, *BAP1* and *BRAF*) have been predicted to differ in their mutational status between intra- and extracranial metastases^6^, whereas another older study had reported an unchanged mutational landscape between both types of metastases^22^. Moreover, intra- and extracranial metastases have also been compared at the gene expression level^22,25^ and at the level of DNA copy number alterations^22^. Additionally, a study that utilized a melanoma brain metastasis model described a neuron-like melanoma phenotype suggesting that the brain micro-environment plays an important role for the adaptation of metastatic melanoma cells to the brain^26^. A more general view of metastases in the brain micro-environment was obtained by a recent single cell transcriptome study that discovered two general archetypes of human brain metastases: a proliferative and an inflammatory archetype, which are shaped by tumor-immune interactions^27^.

In addition, epigenetic alterations have been reported to play an important role for metastases formation^28–30^. Changes of DNA-methylation in promoter regions are well-known to directly alter the expression of affected genes^31^, but also DNA-methylation changes in other genomic regions like enhancers or gene bodies can influence the expression of genes^32–34^. Two studies with melanoma metastases from different patients have revealed a strong heterogeneity between DNA-methylation profiles of intra- and extracranial metastases^35,36^. It is very likely that a large proportion of this epigenetic heterogeneity can be attributed to the fact that metastases from different patients had been compared. To account for this inter-patient heterogeneity, a comparative analysis of genome-wide DNA-methylation profiles of patient-matched intra- and extracranial metastases pairs could help to better characterize DNA-methylation differences to improve the understanding of molecular mechanisms that may contribute to therapy resistance. The potential of such an approach has recently been demonstrated for other omics layers by comparing transcriptomic^25^ and genomic profiles^6^ of patient-matched intra- and extracranial metastases pairs. In addition, paired analyses of DNA-methylation profiles of cell lines of metastatic and primary melanomas identified driver genes that contribute to metastases formation^37,38^.

Here, we analyze DNA-methylation profiles of patient-matched melanoma metastases pairs from intra- and extracranial metastases. To account for the common developmental history of the patient-matched metastases, a Hidden Markov Model (HMM) approach is considered for the personalized analysis of the genome-wide DNA-methylation profiles of patient-specific metastases pairs. The predicted methylation differences are further analyzed in the context of known functional genomic elements, cancer-relevant signaling pathways, and clinical meta-information. Further, the most frequently altered genes across all patients were intensively analyzed based on gene ontologies, pathway information and literature studies to derive a set of potential key pathway candidate genes that could be involved in the manifestation of differences between intra- and extracranial melanoma metastases. Our study contributes to a better characterization of DNA-methylation differences between intra- and extracranial metastases and may help to select potential molecular targets for the development of novel therapeutic strategies for intracranial metastases.

## Results

### Hierarchical clustering of melanoma metastases DNA-methylation profiles suggests a need for a patient-specific analysis

**Figure 1:**
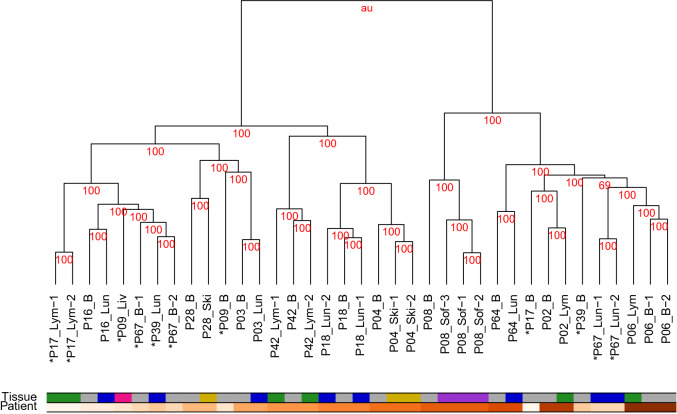
Similarity of methylomes of patient-specific melanoma metastases. Hierarchical clustering of genome-wide methylation profiles of 16 intra- and 21 extracranial metastases from 14 patients (Table 1). Patient-specific metastasis samples are co-clustered together in almost all cases independent of the tissue type in which the metastases were located. This patient-specific rather than tissue-specific clustering of melanoma metastases is given by the order of the metastases labels and illustrated by the patient color gradient below the dendrogram. The samples of four patients that did not cluster together are marked by an asterisk ‘*’. Different tissues from which the metastases were taken are encoded in the metastases labels and highlighted by the tissue color code below the dendrogram (grey: brain, green: lymph node, blue: lung, pink: liver, yellow: skin, purple: soft tissue). The red number above each subcluster in the dendrogram represents the approximate unbiased *p* value (AU value) determined by bootstrapping, where a value of 100 means that the corresponding subcluster was completely stable.

Our data set represents genome-wide methylation profiles of patient-matched intra- and extracranial melanoma metastases of 14 patients (Supplementary Table S1). Extracranial metastases include lymph node, lung, liver, skin and soft tissue (Table 1). For seven patients, multiple samples from histologically different regions were taken from the same metastasis. To obtain a global overview of similarities and differences of the individual metastases, a hierarchical clustering analysis was performed for the measured genome-wide methylomes (Fig. 1). For the vast majority of samples, this clustering showed that metastases of the same patient were much more similar to each other than to metastases of the same tissue from other patients. The hierarchical clustering also revealed two major subclusters. In most cases, all samples of an individual patient were co-clustered in one of both subcluster, except for the patients P17, P39 and P67, whose intra- and extracranial metastases were spread over both major subclusters. Overall, for 4 of 14 patients (P09, P17, P39, P67) their intra- and corresponding extracranial metastasis were not directly co-clustered together. Intracranial, lymph node, and lung metastases, which were available from more than two patients, were present in both major subclusters. Liver and skin metastases were exclusively part of the left major subcluster, whereas soft tissue metastases were exclusively found in the right major subcluster (Fig. 1).

**Table 1:** Patient and sample overview. Each patient developed an intracranial metastasis and an extracranial metastasis in either lung, lymph node, skin, liver or soft tissue.

Patient	Intracranial metastasis	Extracranial metastasis	Treatment
P02	1 sample	1 lymph node sample	Untreated
P03	1 sample	1 lung sample	Untreated
P04	1 sample	2 skin samples	Untreated
P06	2 samples	1 lymph node sample	IM: CTX & ITX (stopped)
P08	1 sample	3 soft tissue samples	IM, EM: CTX & ITX
P09	1 sample	1 liver sample	Untreated
P16	1 sample	1 lung sample	Untreated
P17	1 sample	2 lymph node samples	Untreated
P18	1 sample	2 lung samples	Untreated
P28	1 sample	1 skin sample	IM, EM: CTX & ITX (stopped); IM: WBRT
P39	1 sample	1 lung sample	IM, EM: ITX (stopped)
P42	1 sample	2 lymph node samples	IM: CTX
P64	1 sample	1 lung sample	Untreated
P67	2 samples	2 lung samples	Unknown

Multiple samples of the same metastasis were taken if they showed histologically different regions. The treatment column provides information if and how a patient was treated before the surgical removal of a metastasis.

*IM* Intracranial metastasis, *EM* Extracranial metastasis, *CTX* Chemotherapy, *ITX* Immunotherapy, *WBRT* Whole brain radiotherapy. Treatment of three patients was stopped at least one month before surgery. Metastases of P67 were obtained post mortem.

Further, an additional bootstrap-based stability analysis^39^ of the hierarchical clustering confirmed that the observed trends were very robust. All AU values, except for one, were 100 (Fig. 1). These results clearly indicate that methylomes of patient-specific metastases from intra- and extracranial tissue tend to be more similar to each other than corresponding methylomes of metastases within the same tissue from other patients. Similar results have been observed for gene expression data of melanoma metastases^25^. Thus, motivated by the observed patient-specific clustering of metastases samples, a targeted comparative analysis of the methylomes of each individual metastases pair was performed as next step to predict alterations that distinguish the intra- from the extracranial metastasis.

### Hidden Markov model based analysis of patient-specific melanoma metastases pairs

**Figure 2:**
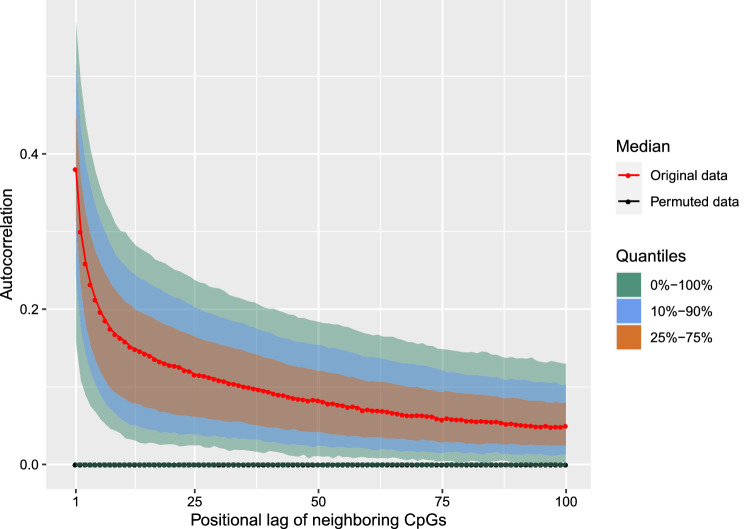
Local similarities of measurements of neighboring CpGs in methylation profiles. Autocorrelations of methylation alterations of patient-specific log$_2$-ratio methylation profiles comparing intra- to corresponding extracranial metastases. Autocorrelations were computed per chromosome for all samples for the originally measured log$_2$-ratio profiles (red) and for randomly permuted log$_2$-ratio profiles (black). Median autocorrelations are plotted on the y-axis against the positional lag of neighboring CpGs within the log$_2$-ratio profiles (x-axis). Corresponding quantiles of the reached autocorrelations are additionally shown for the original profiles (colored bands around the red curve) clearly distinguishing them from the random permutation control model, which was built based on 1000 randomly permuted profiles derived from the originally measured methylomes.

To realize a patient-specific analysis of individual metastases pairs, we first computed genome-wide log$_2$-ratio profiles for each patient-specific metastases pair. Such a log$_2$-ratio profile quantifies for each CpG the pair-specific difference in methylation for the intracranial metastasis relative to the corresponding extracranial metastasis (Supplementary Table S2). These methylation profiles were used to perform an autocorrelation analysis to determine the similarity of methylation alterations in close chromosomal proximity (Fig. 2). The observed strong positive correlations of neighboring measurements motivated the usage of a Hidden Markov Model (HMM) for a comparative analysis of the methylomes of each individual metastases pair, because HMMs model local chromosomal dependencies of measurements to obtain reliable predictions of underlying states (e.g.^40–42^). Therefore, we utilized a specifically adapted three-state standard first-order HMM for the prediction of methylation alterations in individual metastases pairs^40^ (see “Methods” for details). This model classified each CpG in a metastases pair according to its most likely underlying methylation state ensuring a biologically meaningful interpretation: (i) decreased methylation “−”, (ii) unchanged methylation “$=$”, or (iii) increased methylation “$+$” in the intra- compared to the extracranial metastasis. The HMM approach has already been shown to outperform other models for the prediction of altered CpGs in DNA-methylation profiles^40^. The possibility to analyze each patient-specific metastases pair individually makes this approach ideally suited to account for the common developmental history of patient-matched metastases pairs reflected in their DNA-methylation profiles.

**Figure 3:**
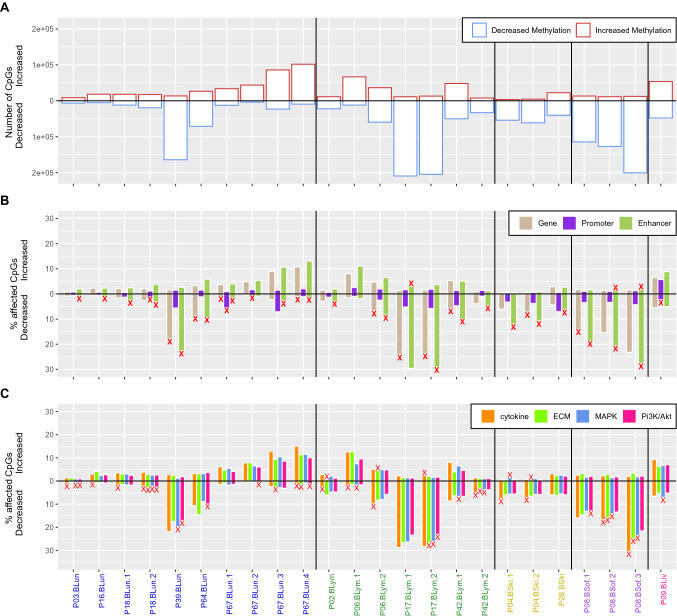
Overview of methylation changes of individual metastases pairs. Patient-specific summary of the HMM-based predictions of the methylation states of individual CpGs. The patient-specific metastases pairs are sorted by the tissue in which the extracranial metastasis occurred (x-axis) with brain versus lung (blue), brain versus lymph node (green), brain versus skin (yellow), brain versus soft tissue (purple), and brain versus liver (pink). (**A**) Total number of differentially methylated CpGs predicted to have a decreased (blue, downward bars from 0) or increased (red, upward bars from 0) methylation in the corresponding patient-specific metastases pair. (**B**) Percentage of CpGs with decreased or increased methylation located in functional genomic elements with a focus on gene bodies, promoters and enhancers for each metastases pair. (**C**) Percentage of increased and decreased methylated CpGs of the most frequently affected signaling pathways. Significant enrichment of a genomic category is marked with a red ‘x’ in subpanels (**B**) and (**C**) (FDR-adjusted *p* value < 0.05).

The HMM-based predictions are summarized in Fig. 3A for each individual metastases pair and further globally provided in Supplementary Table S3. The individual metastases pairs were sorted according to the tissues in which the extracranial metastasis occurred. Most metastases pairs belonged to the group of brain versus lung (10 pairs) followed by brain versus lymph node (7 pairs), brain versus skin (3 pairs), brain versus soft tissue (3 pairs) and brain versus liver (1 pair). On average across all pairs, 7.8% of the measured CpGs showed decreased methylation and 3.4% showed increased methylation in intra- compared to extracranial metastases, whereas the vast majority of 88.8% of CpGs did not differ. Overall, the individual metastases pairs of different patients showed quite different numbers of altered CpGs. Several metastases pairs showed much more CpGs with decreased methylation than CpGs with increased methylation (P08, P39, P17), but there were also some pairs that contained clearly more CpGs with increased than decreased methylation (P67 and pair P06-BLym-1). Other pairs only showed very few methylation differences between the intracranial and the extracranial metastasis and their numbers of CpGs with decreased or increased methylation were comparable (e.g. P03, P09, P16, P18). Generally, these predicted methylation differences were independent of the tissue type of the extracranial metastases. The groups brain versus lung and brain versus lymph node each contained pairs that belong to both extremes. As expected, multiple pairs from the same patient mainly showed similar numbers of CpGs with decreased or increased methylation (P04, P08, P17, P18, P67), but for two other patients (P06, P42) also slightly stronger deviations of their multiple pairs were observed.

All these patients with multiple metastases pairs were further analyzed to determine to which extent the pairs differ in their genome-wide DNA-methylation profiles within the same patient and between patients. Hierarchical clustering in combination with bootstrapping showed that multiple pairs of different histological regions of patient-specific metastases all formed completely stable subclusters for the individual patients (Supplementary Fig. S1A). Multiple pairs of different patients that had their extracranial metastases in the same tissue (brain vs. lung pairs: P18, P67; brain vs. lymph node pairs: P06, P17, P42) were spread across the cluster dendrogram and did not form larger subclusters according to the tissue type in which the extracranial metastases occurred. The observed stable clustering of multiple pairs of different histological regions of patient-specific metastases also remained completely stable when all other patient-matched pairs of patients without multiple samples were included (Supplementary Fig. S1B). This is also supported by the median conformity of the HMM-based predictions of the individual CpG-specific methylation states, which was significantly greater for multiple pairs of the same patient than between pairs of different patients (Wilcoxon rank sum test: $p = 0.0000931$, median conformities: 88.77 vs. 81.96, Supplementary Table S4).

Thus, in combination with the observed co-clustering of patient-specific samples (Fig 1, Supplementary Fig. S1), patient-specific pairs from the same metastasis but different histological regions tend to have very similar alterations of the methylome. Globally, individual patients differ more strongly in their methylomes.

### Functional analysis of predicted patient-specific methylome alterations

To identify which genomic elements were affected by methylation alterations, we analyzed the genome-wide HMM-based predictions of CpGs with decreased and increased methylation for the individual metastases pairs in the context of functional genomic annotations focusing on promoters, gene bodies and enhancers (Fig. 3B). The number of altered CpGs in these functional categories closely followed the observed numbers of altered CpGs in individual pairs (Fig. 3A–B). Significant enrichment of altered CpGs were predominantly found in CpGs located within enhancer regions (21 of 24 pairs, Fig. 3B). Further, 10 of 24 pairs showed enrichment of altered CpGs in gene bodies and 4 of 24 pairs showed enrichment of altered CpGs in promoter regions (Fig. 3B). All these enrichments were observed for CpGs that showed decreased methylation in intra- compared to extracranial metastases, except for P08 and P17, which also showed an enrichment of CpGs with increased methylation in the intracranial metastasis for enhancers. Similar trends were observed for other functional categories including CpG islands, shore, shelf and open sea regions (Supplementary Fig. S2B), where significant enrichment was observed for all categories but most frequently in open sea regions. Further, in accordance with known activation mechanisms^43^, significant enrichment of decreased methylation in intra- compared to extracranial metastases was observed for transposable elements (Supplementary Fig. S2C). Most frequently altered were LTR retrotransposons with significant enrichment in 12 samples, Long Interspersed Nuclear Element (LINE) in 9 samples and DNA transposons in 8 samples.

Further, we also analyzed how CpGs of genes that are part of known cancer-relevant signaling pathways were affected by methylation alterations in individual patient-specific metastases pairs (Fig 3C, Supplementary Fig. S2A). Figure 3C summarizes the results for signaling pathways whose genes were significantly enriched with altered CpGs in eight or more pairs. Genes of the cytokine receptor interaction pathway, for which 14 of 24 pairs showed significantly altered methylation between intra- and extracranial metastases, were most frequently affected (increased methylation in intracranial metastases: P17-BLym-2; decreased methylation in intracranial metastases: P03-BLun, P16-BLun, P18-BLun-1/2, P08-BSof-2/3, P42-BLym-2, P04-BSki-1/2, P06-BLym-1/2,P02-BLym and P67-BLun-4). This enrichment was observed in comparison to all extracranial tissue types, except for liver tissue (P09-BLiv), potentially indicating an important role of the cytokine receptor interaction pathway in the development and manifestation of intracranial metastases. The second most frequently affected pathway was MAPK signaling. Genes of this pathway were significantly enriched in 11 of 24 metastases pairs again with a strong overrepresentation of decreased methylation in intracranial metastases (increased methylation in intracranial metastases: P04-BSki-1; decreased methylation in intracranial metastases: P03-BLun, P06-BLym, P08-BSof-2/3, P09-BLiv, P17-BLym-2, P18-BLun-2, P39-BLun and P42-BLym-1/2). The corresponding enrichment was observed in comparison to all extracranial metastasis tissue types including liver tissue. In addition, genes of the ECM receptor interaction pathway were significantly enriched for altered methylation in 10 of 24 pairs, but this time the proportion of pairs with increased methylation in intracranial metastasis was nearly as large as that of pairs with decreased methylation (increased methylation in intracranial metastases: P02-BLym, P04-BSki-2 and P06-BLym-2; decreased methylation: P08-BSof-2/3, P17-BLym-2, P18-BLym-2, P42-BLym-2 and P67-BLun-3/4). Again, these enrichments were observed in comparison to all extracranial tissue types, except for liver tissue (P09-BLiv). Further, genes of the PI3K/Akt signaling pathway showed a significant enrichment of CpGs with decreased methylation in intracranial tissues for 8 of 24 patient-matched metastases pairs (P03-BLun, P08-BSof-1, P17-BLym-2, P18-BLun-2, P39-BLun, P64-BLun, P67-BLun-2), excluding pairs where the extracranial metastasis occurred in soft tissue or liver. Overall, methylation alterations of CpGs associated with signaling pathway genes were observed for the vast majority of pairs. Only two pairs did not show any significant enrichment of differential methylation in specific signaling pathways (P28-BSki, P67-BLun-2).

### Methylome alterations were not associated with treatment regimens or survival

**Figure 4:**
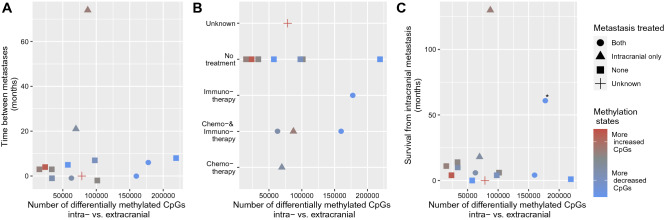
Methylation alterations in the context of clinical information. Visualization of the number of differentially methylated CpGs between intra- and extracranial metastases (x-axis) in the context of different clinical information of each patient (y-axis). Color coding of the plotted symbols defines if the methylation changes include more CpGs with increased methylation (red) or more CpGs with decreased methylation (blue) in intra- compared to extracranial metastases. Shapes of the plotted symbols highlight the treatment status of the individual patients after the occurrence of a metastasis. (**A**) Number of differentially methylated CpGs in the context of the time between both metastases of a patient. Negative time means that the intracranial metastasis occurred before the extracranial metastasis. (**B**) Number of differentially methylated CpGs in relation to treatment status of metastases of individual patients. (**C**) Number of differentially methylated CpGs in relation to survival from intracranial metastasis. Patient P39 was still alive and is marked with an asterisk ’*’.

Available clinical meta-information about the time between surgical resections of patient-matched metastases, received treatments, and survival after the occurrence of the intracranial metastasis of patients (Supplementary Table S5) were considered to analyze whether the predicted methylation alterations between patient-matched pairs of intra- and extracranial metastases were associated with specific clinical characteristics. In a first approach, the total number of HMM-based predicted differentially methylated CpGs was analyzed in relation to each of these three clinical characteristics. The time between the surgical resection of both metastases was less than 10 months for 12 of 14 patients. Within these highly similar time frames, quite different numbers of DNA-methylation alterations were observed ranging from less than 25,000 up to more than 225,000 altered CpGs in our cohort (Fig. 4A). Further, there were no indications that drug treatments of patients after the occurrence of the first metastasis had an impact on the number of altered CpGs (Fig. 4B). In more detail, the patient with the smallest and the patient with the greatest number of altered CpGs were untreated. Patients that received a treatment were located between those extremes overlapping with other untreated patients. In addition, the number or state of altered CpGs did not have an impact on survival from the occurrence of an intracranial metastasis (Fig. 4C). In a second approach, it was analyzed whether the most discriminative CpGs between patient-matched intra- and extracranial metastases differ in their methylation states between untreated and treated patients. This was first done by considering all 14 top-ranking CpGs that either showed decreased or increased methylation in at least 10 of 14 patients (Supplementary Table S6, 1 CpG with increased and 13 CpGs with decreased methylation in intra- compared to extracranial metastases pairs). Overall, the majority of these CpGs showed similar proportions of methylation alterations for untreated and treated patients (Supplementary Fig. S3). In more detail, all three patients that received a treatment against both metastases before surgical resection showed alterations of these top-ranking CpGs. Except for four CpGs, the top-ranking CpGs were also altered in the same manner in at least one of two patients that only received a treatment before the resection of the intracranial metastasis and at least five of eight untreated patients also showed the same alterations of these CpGs. These numbers remained largely stable when the selection criterion was further relaxed to all 661 CpGs that either showed decreased or increased methylation in at least 8 of 14 patients (Supplementary Table S6). Thus, the most discriminative top-ranking CpGs showed rather homogeneous methylation alterations for treated and untreated patients and tend to represent more general marker candidates to distinguish intra- from extracranial metastases.

### Global top-ranking gene candidate analysis across all patients

**Figure 5:**
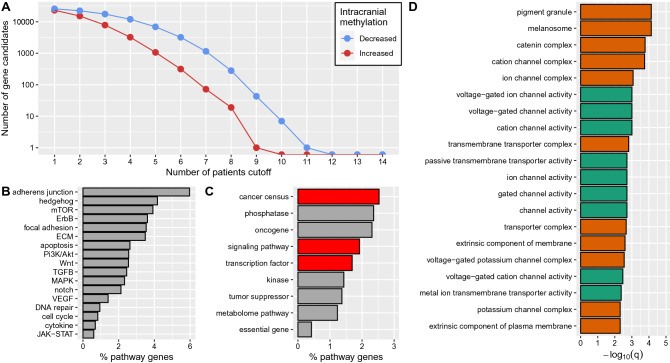
General overview of gene candidates with altered methylation between intra- and extracranial metastases pairs. (**A**) Number of gene candidates with decreased (blue) and increased (red) methylation in intracranial metastases that were identified in at least a specific number of patients (x-axis) in relation to their corresponding extracranial metastases. (**B**–**D**) Analysis of gene annotations of candidate genes that were altered in the same direction in at least 8 patients. (**B**) Percentages of affected genes of specific signaling pathways among the gene candidates that showed differential methylation. (**C**) Percentages of candidate genes in general functional annotation categories. Significantly overrepresented categories are shown in red (FDR-adjusted *p* value < 0.05). (**D**) Top overrepresented gene ontology terms at the significance cutoff of − log$_{10}$(q-value) > 2 representing molecular function (green) and cellular composition (orange).

To further investigate the predicted methylation changes between intra- and extracranial melanoma metastases at the gene level, the most frequently affected genes were determined by accounting for a minimal number of patients in which these genes were altered in the same direction (Fig. 5A). As expected from the heterogeneity of the methylomes across the patients, the number of candidate genes decreased for increasing numbers of patients that had to share a specific gene. Further, the number of candidate genes with decreased methylation was always greater than number of candidate genes with increased methylation, which was also expected because decreased methylation was found to occur more frequently in intra- compared to extracranial metastases (Fig. 3A). In addition, no gene was altered in the same direction in all patients. Therefore, a cutoff of at least 8 patients was considered to characterize the genes that were more frequently altered in more than 50% of patients. The corresponding candidate gene set contained 299 genes of which 280 showed decreased and 19 showed increased methylation in intra- compared to extracranial metastases (Supplementary Table S7). Considering known cancer-relevant signaling pathways, the greatest overlap of these genes was observed for the adherence junction pathway (6% of candidate genes) followed by hedgehog (4.1%) and mTOR (3.9%) signaling (Fig. 5B). In addition, for MAPK, PI3K/Akt, and ECM signaling, which were previously found to be significantly enriched in the individual analysis of the patient-specific metastases pairs, about 2.8% of the candidate genes overlapped with each of these three pathways. However, for the cytokine signaling pathway, which was most frequently enriched in the individual analysis of the metastases pairs, only an overlap of about 1% was observed within this integrative analysis of all patients. Thus, genes of the cytokine signaling pathway are affected by methylation changes in individual patients, but the majority of affected genes tended to differ between the patients. Still, important cytokine signaling genes such as *GDF15* with decreased intracranial methylation and *BMP4* with increased intracranial methylation were altered in at least 8 patients. Overall, the candidate gene set was significantly enriched for known cancer census genes (e.g. *SGK1*, *MECOM*, *PRKCB*) and known transcription co/factors (e.g. *CACNA2D3*, *BMP4*) (Fig. 5C).

In addition to this basic annotation analysis, a gene ontology (GO) enrichment analysis was performed for the candidate gene set (Fig. 5D). Interestingly, functional terms related to channel and transporter activities (cation channel activities, voltage-gated channel activities) were among the top-ranking GO molecular function terms. Further, cellular components like ion and cation channel complexes, pigment granule and melanosomes were among the most enriched GO component terms. These findings could indicate a connection to the neuron-like melanoma phenotype that has been described for intracranial metastases^26^. Moreover, the enrichments of GO terms in potential relation to a neuron-like phenotype were even stronger for a larger gene candidate set of 1222 genes that were altered in the same direction in at least 7 of 14 patients (Supplementary Fig. S4).

Finally, genomic regions that had been reported to be altered in their DNA-methylation in metastatic compared to matched primary melanoma cell lines^37,38^ were analyzed for their methylation behavior in our patient-matched intra- and extracranial metastases. For the study by Vizoso et al.^37^, 2410 of 2620 CpGs that differed in methylation between metastatic and primary melanoma cell lines were shared with our methylome data set (Supplementary Table S8). These CpGs were associated with 1549 genes that behaved in terms of methylation alterations between patient-matched intra- and extracranial melanoma metastases very similar to the global analysis of all genes meaning that clearly more of these genes showed decreased rather than increased methylation in intra- compared to extracranial metastases (Supplementary Fig. S5A). Further, the top candidate gene *TBC1D16*, which was found to have lost its methylation in metastatic cell lines to trigger a metastatic cascade^37^, also showed a decreased methylation in the intra- compared to the extracranial metastasis for 10 of 14 patients. Thus, *TBC1D16* could therefore also potentially have an important role for the development of intracranial metastases. A similar analysis was done for the 23 genes that were associated with the 10 hyper- and the 65 hypomethylated regions from Chatterjee et al.^38^ that had been identified to distinguish metastatic from primary matched melanoma cell lines (Supplementary Table S9), but none of these genes showed altered methylation in more than five patients comparing patient-matched intra- and extracranial metastases (Supplementary Fig. S5B).

### Methylation alterations of frequently altered signaling pathway genes distinguish intra- from extracranial metastases

**Figure 6:**
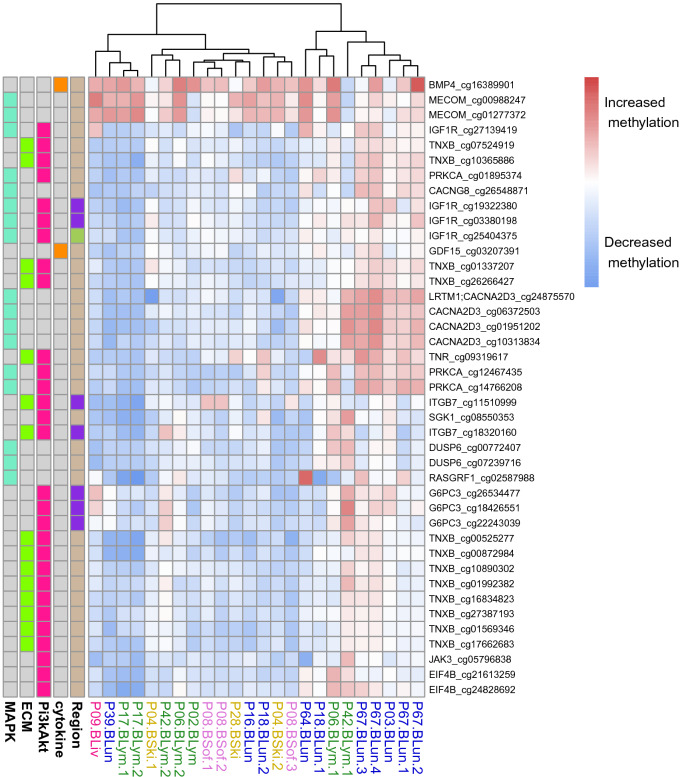
Heatmap of log$_2$-ratios of methylation levels of CpGs associated with the 17 signaling pathway gene candidates that best distinguish intra- and extracranial melanoma metastases. The log$_2$-ratios quantify the methylation changes in the intra- compared to the corresponding extracranial metastasis for each patient-matched metastases pair. Color coding of the sample names below the columns of the heatmap represents the tissues in which the extracranial metastases occurred (blue: lung, green: lymph node, yellow: skin, purple: soft tissue, pink: liver). Gene names associated with individual CpGs are shown along the rows at the right side of the heatmap. The gene names are further extended by the underlying CpG identifiers. The location of each CpG relative to its associated gene are displayed at the left side of the heatmap along the rows distinguishing between promoter (purple), gene body (brown) or enhancer (green) regions. Specific pathway memberships of the candidate genes are also shown at the left side.

The initial candidate gene set was further reduced to the 17 included signaling pathway genes that were altered in the same direction in at least 8 of 14 patients to enable a focused analysis of the selected cytokine, ECM, MAPK, and PI3K/Akt signaling pathway genes. Corresponding methylation changes between intra- and extracranial metastases pairs are shown in Fig. 6 for associated gene-specific CpGs. The vast majority of these CpGs was located in gene bodies (88.9%) followed by clearly smaller proportions in promoter (6.7%) and enhancer regions (4.4%). The heatmap in Fig. 6 further represents a strong separation of the metastases pairs into two major subclusters, where the smaller subcluster on the right side (8 pairs) mainly shows increased methylation levels in intracranial metastases in comparison to the larger subcluster on the left side (14 pairs). These two major clusters were not separated by the type of tissue in which the extracranial metastases occurred, but all four metastases pairs of patient P67 were include in the right subcluster. This contributed mainly to the size of the right subcluster, but also samples of other patients (P03, P06, P18, P42) were included. Nevertheless, other samples of these four patients were part of the left subcluster indicating that methylation alterations of the candidate genes can differ between distinct histological regions of patient-specific metastases. Further, consistent methylation changes were observed for multiple CpGs that were associated with the same gene. This is reflected by a nearby positioning of gene-specific CpGs in the clustering. Only three CpGs of two candidate genes (*BMP4*, *MECOM*) tended to show an increased methylation across all metastases pairs, whereas the other CpGs showed much stronger methylation differences between both clusters.

In addition, the 17 signaling pathway candidate genes were considered for an in-depth annotation analysis and literature search to summarize potential functions and connections to cancer and other diseases (Table 2). Overall, the 17 candidate genes can be mainly assigned to four functional annotation groups: (i) genes involved in cell-cell signaling (*PRKCA*, *CACNG8*, *RASGRF1*, *CACNA2D3*), (ii) genes involved in cell growth (*DUSP6*, *IGF1R*, *GDF15*, *BMP4*), (iii) genes involved in cell death (*SGK1*, *MECOM*), and (iv) genes involved in the regulation of cell adhesion (*TNXB*, *ITGB7*, *TNR*). Further, strong literature connections to cancer were found for ten candidate genes. Most of these genes were associated with cancers of metabolic systems (*DUSP6*, *SGK1*, *BMP4*, *RASGRF1*, *GDF15*) followed by breast cancer (*CACNA2D3*, *ITGB7*), cancers of the central nervous system (*PRKCA*), or leukemia (*MECOM*, *JAK3*). Some of the candidate genes are also known to play a role in neuronal diseases like Alzheimer’s disease (*EIF4B*), schizophrenia (*CACNG8*) or Parkinson’s disease (*TNR*).

**Table 2:** Top differentially methylated signaling pathway genes distinguishing intra- from extracranial metastases.

Gene	Methylation state in intracranial metastases	Number of patients	Affected region	Affected CpG identifiers	Biological process	Role in cancer/other diseases	References
TNXB	Decreased	10	Body	cg00525277, cg00872984, cg01337207, cg01569346, cg01992382, cg07524919, cg10365886, cg10890302, cg16834823, cg17662683, cg26266427, cg27387193	Cell adhesion	Ehlers-Danlos syndrome	^44^
PRKCA	Decreased	9	Body	cg01895374, cg12467435, cg14766208	Cell signaling	Glioma	^45^* ,^46^*
BMP4	Increased	9	Body	cg16389901	Cell growth	Gastric cancer	^47^*
DUSP6	Decreased	9	Body	cg00772407, cg07239716	Cell growth	Pancreatic, gastric cancer	^48^*,^49^*
SGK1	Decreased	9	Body	cg08550353	Cell death	Epilepsy, schizophrenia, Alzheimers disease, Colon cancer, gastric cancer	^50,51^, ^52^*, ^53^*
ITGB7	Decreased	9	Promoter	cg11510999, cg18320160	Cell adhesion	Breast cancer	^54^*
EIF4B	Decreased	9	Body	cg21613259, cg24828692	Translation regulation	Alzheimers disease	^55^
G6PC3	Decreased	9	Promoter	cg18426551, cg22243039, cg26534477	Metabolism	Neutropenia	^56,57^
CACNA2D3	Decreased	8	Body	cg01951202, cg06372503, cg10313834, cg24875570	Cell signaling (Ca2+)	Breast cancer metastases	^58^*
CACNG8	Decreased	8	Body	cg26548871	Cell signaling (Ca2+)	Schizophrenia	^59^
RASGRF1	Decreased	8	Body	cg02587988	Cell signaling	Colorectal cancer	^60^*
GDF15	Decreased	8	Body	cg03207391	Cell growth	Colorectal cancer	^61^*
IGF1R	Decreased	8	Body, enhancer, Promoter	cg03380198, cg19322380, cg25404375, cg27139419	Cell growth		
MECOM	Increased	8	Body	cg00988247, cg01277372	Cell death	Leukemia	^62^*
TNR	Decreased	8	Body	cg09319617	Cell adhesion	Parkinsons disease	^63^
JAK3	Decreased	8	Body	cg05796838	Immune response	Leukemia	^64^*
LRTM1	Decreased	8	Body	cg24875570	Transmembran domain		

The table represents in-depth information about the 17 signaling pathway genes that contained at least one CpG whose methylation level was altered in the same direction in at least 8 of 14 patients. The observed methylation change in intracranial metastases is reported in the column ‘Methylation state in intracranial metastases’ along with the number of patients whose metastases pair showed the same behavior provided in the column ‘Number of patients’. The affected region of a gene is provided in the column ‘Affected region’, affected CpGs are listed in the column ’Affected CpG identifiers’, and corresponding gene functions from GeneCards^65^ and Uniprot^66^ are summarized in the column ’Biological process’. Results of an in-depth literature analysis in relation to cancer and other diseases are summarized in the column ‘Role in cancer / other diseases’. Relevant references are listed in the column ’References’. References that indicate associations of a specific gene in relation to cancer are marked with an asterisk ‘*’.

### Methylation alterations of frequently altered signaling pathway genes were associated with expression changes between intra- and extracranial metastases

**Figure 7:**
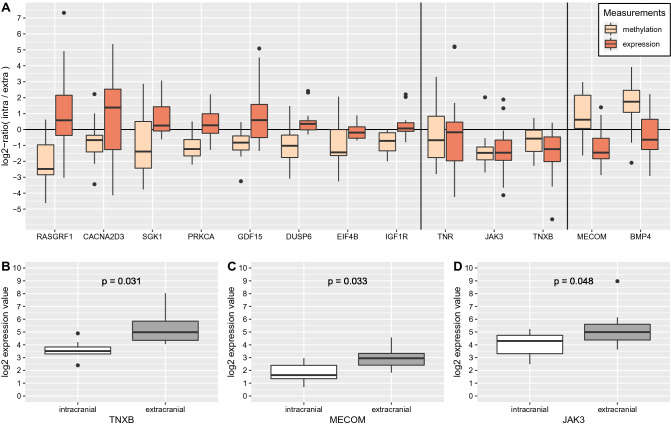
Expression behavior of selected signaling pathway gene candidates with altered gene body methylation that best distinguish intra- and extracranial melanoma metastases based on methylation data. (**A**) Boxplots representing gene body methylation differences (light brown) and gene expression differences (red) based on log$_2$-ratios comparing gene-specific measurements of intra- to extracranial metastases considering available data of eight patients. Gene names are shown below the boxplot pairs. Two vertical black lines were inserted into the plot to enable a better visual separation of the genes into three classes that differ in their methylation and expression behavior of the assigned genes. (**B**–**D**) Boxplots of intra- and extracranial expression levels of the three genes with the greatest expression difference from zero in subpanel (**A**). The three genes were selected at the FDR-adjusted *p* value cutoff of 0.05. *P* values were obtained by paired t-tests comparing gene-specific expression levels of the patient-matched intra- and extracranial metastases pairs.

The identified 17 signaling pathway genes that best distinguished intra- from extracranial metastases predominantly showed alterations of gene body methylation (Fig. 6, Table 2). To analyze if and how these gene body methylation alterations might be associated with the expression of these genes, normalized gene expression measurements of patient-matched metastases from 8 of the 14 considered patients of our cohort were available for a systematic evaluation of 13 genes (Supplementary Table S10). A joint representation of these gene expression data together with the corresponding gene body methylation data suggested a separation of these genes into three more general data relationship categories (Fig. 7A): (i) the majority of genes with decreased gene body methylation showed a trend towards increased expression in intra- compared to extracranial metastases (8 of 11 genes: *CACNA2D3*, *DUSP6*, *EIF4B*, *GDF15*, *IGF1R*, *PRKCA*, *RASGRF1*, *SGK1*), (ii) the other three genes with decreased gene body methylation showed a trend towards decreased expression in intra- compared to extracranial metastases (3 of 11 genes: *JAK3*, *TNR*, *TNXB*), and (iii) both genes with increased gene body methylation (*BMP4*, *MECOM*) showed a trend towards decreased expression in intra- compared to extracranial metastases. Further, a ranking of these 13 genes according to their strengths of expression differences between patient-matched intra- and extracranial metastases showed that especially *JAK3*, *MECOM*, and *TNXB* differed strongly in their expression between intra- and extracranial metastases (Fig. 7B–D, FDR-adjusted *p* value < 0.05). Thus, this validation study indicates that gene body methylation alterations could be functionally linked to expression changes of affected genes and may thereby contribute to differences between intra- and extracranial melanoma metastases.

## Discussion

The development of intracranial melanoma metastases is a crucial factor for survival of melanoma patients. Therefore, the identification of molecular differences between intra- and extracranial metastases is a very important step towards a better characterization of molecular alterations that may contribute to treatment resistance of intracranial melanoma metastases. Since epigenetic alterations could potentially be involved in the establishment and manifestation of molecular differences between intra- and extracranial metastases^22,67^, we have focused on an in-depth analysis of genome-wide methylomes of patient-matched pairs of intra- and extracranial melanoma metastases. The initial hierarchical clustering analysis of these methylomes clearly showed that the patient-specific origin of the individual metastases had a stronger influence on the methylome than the organ in which a metastasis was found. This was also confirmed by an additional hierarchical clustering of patient-matched metastases pairs from patients for which samples of different histological regions of patient-specific metastases were available. Such an impact of the patient-specific ancestry of metastases has also recently been reported for gene expression profiles of melanoma metastases^25^. To account for this, we used a well-established three-state HMM approach^40^ to perform a patient-specific analysis of each individual metastases pair. This enabled us to predict the most likely underlying biologically plausible methylation status for each CpG of each individual metastases pair.

We observed a general trend towards decreased methylation in intra- compared to extracranial melanoma metastases at the level of individual CpGs and at the level of affected genes. This loss of methylation was found for most patient-matched pairs, but several others also showed a more balanced behavior between losses and gains of methylation or a clear increase in methylation in intracranial metastases. Thus, the potential role of methylome alterations in the development of melanoma metastases seems to be very complex varying from patient to patient. Still, the more frequently observed global reduction of methylation levels in intracranial metastases may represent a specific adaptation mechanism of those metastases to the specific micro-environment of the brain. Such an adaptation would also be supported by the general fact that a loss of methylation can play important roles in tumor formation and progression by influencing genomic stability and gene expression^68–70^.

As suggested by the hierarchical clustering and as further identified by the HMM-based predictions of the methylation states of individual CpGs, the methylomes of the individual metastases were very patient-specific. Additional comparisons of the HMM-based predictions of methylome alterations of multiple metastases pairs from the same patient to metastases pairs of other patients clearly confirmed this patient-specific alterations of the methylomes. This observation is in good accordance with findings for methylomes of melanoma brain metastases^35^. Due to the strong patient heterogeneity and the small number of patients, it is not very surprising that no associations of the predicted methylome alterations could be found in relation to patient survival or treatment. Since the patient-specific metastases pairs within the cohort were selected by focusing on patients whose metastases occurred in comparable time frames, it is only possible to say that from relatively few up to many methylation alterations (1.8–26.2% of all CpGs of a patient were differentially methylated) may occur in similar time frames between the extra- and intracranial metastasis. Another important observation was that the most discriminative CpGs that distinguished intra- from extracranial metastases tended to show largely homogeneous methylation alterations for treated and untreated patients suggesting that they could represent more general marker candidates independent of the treatment status.

Further, a functional annotation analysis of the predicted methylome alterations showed several interesting patterns across the patient-matched metastases pairs. Differential methylation was observed on a genome-wide scale and functional genomic elements like promoters, enhancers and gene bodies were significantly affected. Additionally, a loss of methylation in intracranial metastases was revealed for transposon families, most dominantly for long terminal repeats (LTR) transposons (12 of 24 pairs). Such a hypomethylation can trigger a reactivation of transposons, which could contribute to additional DNA mutations^71^ and may thereby influence cancer development^72^. At the level of cancer-relevant signaling pathways, mainly genes of MAPK, PI3K/Akt, ECM and cytokine signaling were frequently affected by a loss of methylation in intracranial metastases. MAPK and PI3K/Akt signaling, which are involved in the regulation of cell proliferation and survival, are already known for their important roles in the regulation of proliferation of melanoma brain metastases^5–7,22,23^. Both pathways are targeted by drugs such as BRAF and PI3K inhibitors for the treatment of metastatic melanoma^24,73–76^. The cytokine signaling pathway plays an important role in immune responses and is further known to be involved in tumor progression^77,78^. Interestingly, 13 of all 24 metastases pairs showed an enrichment of decreased methylation alterations of cytokine pathway genes indicating that this pathway could be involved in the manifestation of differences between patient-specific intra- and extracranial metastases. However, cytokine pathway genes were not significantly enriched among the candidate genes that shared the same methylation state in at least 50% of patients. Thus, different cytokine pathway genes are affected by methylation changes in individual patients.

Considering the candidate gene set of genes that were observed to have the same methylation status in at least 50% of patients, genes were often associated with neuron-type ontology terms like synapses and voltage-gated channels. Since the analyzed regions of the metastases were marked by an experienced pathologist, only a minimal contamination by normal brain cells is expected, which cannot explain the observed strong neuron-type associations. Thus, the methylation alterations in intracranial metastases may have contributed to a brain-like phenotype, supporting similar previous findings^26,79^. Further, some of these individual gene candidates also showed frequent associations with neurodegenerative diseases. This might indicate shared adaptations to the brain microenvironment.

In addition, the candidate gene set was reduced to known cancer signaling pathway genes to enable an in-depth analysis of the resulting 17 candidate genes (Table 2). Corresponding hierarchical clustering of the gene-associated CpGs revealed two major clusters (Fig. 6). Both clusters shared three CpGs with increased methylation in intracranial metastases that belonged to two genes, *BMP4* and *MECOM*. *BMP4* is a growth-factor that also plays an essential role in neurogenesis^80^. The observed increased gene body methylation of *BMP4* in intra- compared to extracranial metastases was associated with decreased expression in intracranial metastases, which may contribute to differences in cell growth and proliferation between intra- and extracranial metastases. *MECOM* is a well-known oncogene involved in cell proliferation and apoptosis and reported to be overexpressed in e.g. leukemia^62,81^, ovarian cancer^82^, and glioblastoma^83^. Again, the observed increased gene body methylation of *MECOM* was associated with decreased expression in intracranial metastases, which may contribute to differences in metastasis manifestation and progression between intra- and extracranial metastases. All other CpGs differed in their methylation between both clusters. The larger cluster showed mostly decreased methylation of CpGs in intra- compared to extracranial metastases, whereas the smaller clustered showed a more heterogeneous methylation behavior of the individual CpGs including increased and decreased methylation in intracranial metastases. In addition, most of the gene-associated CpGs (88.9%) were located within the gene body region, whereas only 6.7% were associated with the gene promoter region. Thus, alterations of gene body methylation patterns seems to be an important feature to distinguish intra- and extracranial metastases pairs. In addition to our observed and other potentially existing alterations of gene expression levels of affected genes^34^, such gene body alterations may also have an impact on splicing^84^. However, it is important to note that the observed difference in the proportions of gene body and promoter methylation alterations is also influenced by the fact that the underlying microarray represents more CpGs within gene body regions (56.6%) than in promoter regions (13.7%). Nevertheless, our in-depth gene annotation analyses of the most frequently altered signaling pathway genes suggest that intra- and extracranial metastases potentially differ in cell-cell signaling, cell adhesion, cell growth, and cell death. Some of the identified genes may therefore also contribute to known differences in treatment efficiencies for intra- and extracranial metastases.

Further, an analysis of genomic regions that have previously been reported to be altered in methylation between matched metastatic and primary melanoma cell lines^37^ showed that the genes that were associated with these regions can also be altered in their methylation in patient-matched pairs of intra- and extracranial metastases. Especially the top-ranking candidate gene *TBC1D16* found by Vizoso et al.^37^ also showed decreased methylation in patient-matched intra- compared to extracranial metastases pairs in more than 70% of patients. Thus, the loss of methylation of *TBC1D16* in metastatic melanoma cell lines that has been reported to trigger a metastatic cascade^37^ might also be involved in the manifestation of differences between intra- and extracranial metastases.

Moreover, gene body methylation alterations of signaling pathway genes that best distinguished intra- from extracranial metastases were directly associated with expression changes. A small gene expression validation data set enabled to analyze the expression behavior of 13 top-ranking signaling pathway genes based on eight patients from our cohort. Three data relationship categories for associations between gene body methylation and expression changes were found. In most cases, decreased gene body methylation was associated with an increased expression of the affected gene (*CACNA2D3*, *DUSP6*, *EIF4B*, *GDF15*, *IGF1R*, *PRKCA*, *RASGRF1*, *SGK1*), but there were also three genes with decreased gene body methylation that were associated with decreased expression in intra- compared to extracranial metastases (*JAK3*, *TNR*, *TNXB*). Both genes with increased gene body methylation were associated with decreased expression in intra- compared to extracranial metastases (*BMP4*, *MECOM*). Thus, gene body methylation can potentially influence the expression of affected genes, but potential relationships between methylation and expression alterations were complex and gene-specific. However, *TNXB*, *MECOM*, and *JAK3* were the three top-ranking genes with altered gene body methylation that significantly differed in their expression between patient-matched intra- and extracranial metastases. Since these three genes are known to be involved in the regulation of cell adhesion, cell death, or immune responses (Table 2), gene body methylation alterations of these genes may contribute to the observed expression differences and thereby potentially be involved in the establishment and manifestation of differences between intra- and extracranial metastases.

Overall, our study represents the first personalized genome-wide analysis of DNA-methylation profiles that contributes to an improved and more detailed characterization of epigenetic differences between patient-matched intra- and extracranial melanoma metastases. Such a personalized analysis accounts for patient heterogeneity and represents an important initial step towards a better stratification of individual patients with melanoma metastases. However, our methylome alteration findings are based on 14 patients and should therefore be further validated in future studies with patient-specific metastases pairs of additional patients. The identified altered signaling pathways and the selected candidate genes can provide a basis for further experimental studies to analyze how these findings are impacting on gene and protein expression on a genome-wide scale to determine potential targets for the development of improved therapies for intracranial metastases of melanomas.

## Methods

### Methylome data of melanoma metastases

Genome-wide DNA-methylation profiles of paired intra- and extracranial melanoma metastases from one of our prior projects were used to determine patient-specific methylome alterations (Gene Expression Omnibus (GEO): GSE203152). The considered part of this data set comprised methylation profiles of 37 metastases samples from 14 melanoma patients. Each of these patients had an intracranial and an extracranial melanoma metastasis during the course of the disease. For seven patients, multiple samples from histologically different regions of the same metastasis were considered. Detailed information about the different metastases types and clinical data are summarized in Table 1. The considered methylation profiles were measured on IlluminaHuman Methylation EPIC arrays followed by preprocessing in R (R packages minfi, IlluminaHumanMethylation450kmanifest and IlluminaHumanMethylation-EPICanno.ilm10b2.hg19^85^) in combination with normalization by the R function preprocessFunnorm utilizing the sex of patients. The CpGs that were represented by probes on the microarray were further filtered for polymorphic and off-target probes based on data from^86^. Log$_2$-ratios comparing methylated to unmethylated hybridization signals were computed for each of the 836,320 CpGs in each sample (Supplementary Table S1).

### Hierarchical clustering of melanoma metastases

The sample-specific CpG-log$_2$-ratios comparing methylated to unmethylated hybridization signals (Supplementary Table S1) were used to perform a hierarchical clustering analysis for the methylomes of the individual metastases using the R package pvclust^39^. Manhattan distance was chosen as distance metric between individual samples due to the high dimensionality of the data^87^ in combination with Ward’s minimum variance linkage method (ward.D2)^88^ for clustering. The pvclust approach was used with 10,000 bootstrapping repetitions in order to analyze the stability of the clustering. This stability was quantified using the approximate unbiased *p* value (AU value). This value ranges from 0 to 100, where a larger value indicates greater stability. The same bootstrap approach was also considered to evaluate the robustness of the hierarchical clusterings of the patient-specific metastases pairs to determine similarities and differences of multiple metastases pairs of different histological regions of the same patient or between patients (Supplementary Fig. S1).

### HMM-based analysis of patient-specific metastases pairs

To obtain a log$_2$-methylation ratio for each CpG of a patient-specific metastases pair, the obtained CpG-specific methylation measurement of the extracranial metastasis was subtracted from the corresponding intracranial metastasis methylation measurement. The log$_2$-methylation ratios were then ordered by the chromosomal locations of the individual CpGs to obtain a genome-wide DNA-methylation profile for each patient-specific metastases pair (Supplementary Table S2). The underlying log$_2$-ratios specify the methylation difference between intra- and extracranial metastases for each patient and contain the following information: (i) log$_2$-ratios clearly less than zero indicate decreased methylation in the intracranial metastasis, (ii) log$_2$-ratios about zero indicate unchanged methylation, and (iii) log$_2$-ratios clearly greater than zero indicate increased methylation in the intracranial metastasis in comparison to the corresponding patient-matched extracranial metastasis. Since neighboring CpGs in close chromosomal proximity had similar measurements (Fig. 2), we considered a Hidden Markov Model (HMM) that can utilize such local dependencies for the analysis of the individual metastases pairs. In more detail, we analyzed the resulting chromosome-specific log$_2$-ratio methylation profiles of a patient-specific metastases pair by a standard first-order three-state HMM with state-specific Gaussian emission densities specifically developed for the analysis of DNA-methylation profiles^40^. This HMM uses three states to classify each CpG according to its methylation level: (i) state “−” models CpGs with decreased methylation in the intracranial compared to the corresponding extracranial metastasis, (ii) state “$=$” models CpGs with unchanged methylation levels, and (iii) state “$+$” models CpGs with increased methylation in the intracranial compared to the corresponding extracranial metastasis. Motivated by the distribution of the log$_2$-ratios for the comparison of patient-matched intra- and extracranial metastases (Supplementary Fig. S6), initial means of the state-specific Gaussian emission distributions were set to − 3, 0, and 3 and corresponding initial standard deviations were set to 0.3, 0.5, and 0.3 for the states “−”, “$=$”, and “$+$”, respectively. In addition, initial state probabilities were set to 0.1 for states “−” and “$+$” and to 0.8 for state “$=$” to account for the fact that the vast majority of CpGs tended to be unchanged. Next, the initial HMM was trained utilizing all patient-matched log$_2$-ratio methylation profiles using the Bayesian Baum-Welch algorithm that allows to integrate prior knowledge about expected methylation differences into the training. To realize this, a grid search was used to determine hyperparameter settings for the state-specific Gaussian emission densities to enable a biologically meaningful interpretation of the states of the trained HMM. This search was used to minimize the number of potentially wrongly classified CpGs with negative log$_2$-ratios assigned to state “$+$” or CpGs with positive log$_2$-ratios assigned to state “−” while maximizing the total number of CpGs assigned to state “$+$” or “−” to obtain a clear separation of methylation alterations. A good separation between reduced, unchanged, and increased methylation levels of CpGs was reached by setting the values of the scale parameter (scaleMeans) to 1e+06, 1e+03, 1e+06 and those of the shape parameter (shapeSds) to 5e+05, 10, 5e+05 for the states “−”, “$=$”, and “$+$”, respectively. All other hyperparameters were kept at the predefined standards. The training of the HMM on all patient-matched log$_2$-ratio methylation profiles ensured that the predictions of the HMM remained comparable across the patients. The training took 98 iteration steps that were done in about 40 minutes on a standard laptop (CPU: 1.80 GHz, 8GB RAM) using the basic HMM implementation based on the Java JAR file from^89^. State-posterior decoding was used to assign each CpG in a patient-specific log$_2$-ratio methylation profile to its most likely underlying methylation state (Supplementary Table S3).

### Functional annotation analysis of HMM-based decodings of genomic CpGs

Each CpG was annotated according to the overlap of its genomic location with the location of functional genomic features. Promoter and gene annotations were obtained from the IlluminaHuman Methylation EPIC arrays regulation features resource^90^. CpG-island annotations were obtained from the R package IlluminaHuman Methylation EPIC Cando^85^. Enhancer annotations were obtained by the R package annotatr utilizing annotations from FANTOM5. Signaling and metabolic pathway genes and cancer-related annotations of genes were taken from^91^. Transposon annotations were obtained from UCSC genome browser^92^. Statistical significance for enrichment of increased and decreased methylated CpGs in a specific functional category was tested using a one-sided Fisher’s exact test (R function fisher.test). All categories with a FDR-adjusted *p* value (R function p.adjust^93^) below 0.05 were considered to be significantly enriched.

### Search for associations of methylation alterations with clinical meta-information

Clinical meta-information about time between patient-matched metastases, treatment and survival were available for most patients (Supplementary Table S5). These data were utilized to search for potentially existing associations with genome-wide methylation alterations that were predicted by the HMM for each patient-matched metastases pair. Therefore, the number of CpGs with increased (state “$+$”) and decreased (state “−”) methylation in the intra- compared to the extracranial metastasis was computed for each pair. Based on that, the number of differentially methylated CpGs was determined for each pair by summing up both counts. For multiple metastases pairs from the same patient, average numbers of increased and decreased counts were considered. Further, a score was defined to quantify for each patient if its intracranial metastasis had more increased or more decreased methylation levels of CpGs compared to its corresponding extracranial metastasis. This was done for each patient by dividing the difference between the numbers of CpGs with increased and decreased methylation by the corresponding sum of differentially methylated CpGs. The resulting score is always in the range of [-1, 1]. Negative score values specify that more CpGs with decreased than with increased methylation levels in the intracranial metastasis were observed for a patient and positive score values specify the opposite. To identify potentially existing trends, scatter plots were used to plot the number of differentially methylated CpGs against the time between both metastases, treatment groups and the time to death from the diagnosis of the intracranial metastases.

### Selection of differentially methylated gene candidates

To identify gene candidates that were frequently affected by DNA-methylation changes, all CpGs within gene-specific promoters, enhancers and gene bodies were ranked across all patients based on their numbers of predicted decreased or increased methylation by the HMM (Supplementary Table S7). For patients with multiple metastases pairs, each CpG of a patient was combined using a majority vote based on decreased and increased predictions. Note that in this context 0.07% of all CpGs had an equal number of decreased and increased methylation prediction and were therefore not decidable and considered as unchanged methylation to exclude them from the analysis. This allows to consider each patient only once in the global CpG-ranking. Promoters, enhancers and gene bodies were considered for the ranking of genes, because they were found to be significantly enriched in the genome-wide analysis of individual DNA-methylation profiles (Fig. 3B) and it is known that these genomic elements are important for the regulation of gene expression^94–96^. Based on the ranking of the gene-associated CpGs, first a global set of top-ranked genes was determined by selecting each gene that had at least one corresponding gene-specific CpG predicted to show decreased or increased methylation in more than 50% of the patients (at least 8 of 14 patients, Supplementary Table S7). This resulted in 299 gene candidates. A gene ontology enrichment analysis by clusterProfiler^97^ was done for these genes to obtain a global characterization of molecular features that differ between intra- and extracranial metastases. Therefore, gene names were mapped to ENTREZ identifiers using the function bitr of clusterProfiler (84% of genes present on the EPIC microarray and 88% of the top-ranked genes were mappable). In addition, an in-depth annotation and literature analysis was done for the 17 annotated signaling pathway genes of the gene candidates using Uniprot^66^, GeneCards^65^ and Pubmed (https://pubmed.ncbi.nlm.nih.gov/) to obtain more detailed insights into potentially existing differences between intra- and extracranial metastases.

### Gene expression analysis of selected differentially methylated gene candidates

Gene expression data for 13 of 17 selected signaling pathway candidate genes with altered methylation were available from a companion project to analyze if and how DNA-methylation alterations influence the expression of these genes. This validation data set comprised processed patient-matched RNA-seq gene expression measurements of intra- and extracranial melanoma metastases that were available for 8 of 14 patients of our DNA-methylation cohort (Supplementary Table S10). Gene expression log$_2$-ratios were computed for each of the 13 genes for each metastases pair by subtracting the normalized gene-specific log$_2$-expression levels of the extracranial metastasis from the corresponding log$_2$-expression levels of the intracranial metastasis. These log$_2$-ratios enable to characterize the expression behavior of each gene: (i) values clearly less than zero indicate reduced expression, (ii) values about zero indicate unchanged expression, and (iii) values clearly greater than zero indicate increased expression in intra- compared to extracranial melanoma metastases. To determine for each gene if its expression was significantly different from zero, a t-test (R function t.test) was used and the *p* values of all t-tests were adjusted for multiple testing by computing FDR-adjusted *p* values (R function p.adjust^93^). This resulted in three genes with FDR-adjusted *p* values less than 0.05 (*JAK3*, *MECOM*, *TNXB*) for which an additional direct comparison of the patient-matched intra- and extracranial expression levels was done by a paired t-test. This additional analysis accounted equally for each of the eight patients by considering average intra- or extracranial gene expression levels for patients for which measurements of multiple regions of the same metastasis were available (P04, P06, P08, P18, P42).

### Ethics declarations, approval for human experiments, and consent to participate

The utilization of FFPE tumor material for the methylome and expression analyses was approved by the ethics committee of the University of Dresden (EK 48022018). The studies were conducted in accordance with the Declaration of Helsinki. All experiments were performed in accordance with relevant named guidelines and regulations. Informed consent for the usage of the tumor material was obtained from all included patients.

## Supplementary Information

Supplementary Information 1.

Supplementary Information 2.

Supplementary Information 3.

Supplementary Information 4.

Supplementary Information 5.

Supplementary Information 6.

Supplementary Information 7.

Supplementary Information 8.

Supplementary Information 9.

Supplementary Information 10.

Supplementary Information 11.

Supplementary Information 12.

Supplementary Information 13.

Supplementary Information 14.

Supplementary Information 15.

Supplementary Information 16.

## Data Availability

Raw DNA-methylation data are available from Gene Expression Omnibus (GSE203152). Processed DNA-methylation profiles of individual metastases are provided in Supplementary Table S1. DNA-methylation profiles of patient-matched metastases pairs comparing intra- to extracranial metastases are provided in Supplementary Table S2. R code of the performed study is freely available from GitHub under https://github.com/TheresaKraft/MelBrainSys_methylation.
