# Supplementary material for: Patient-specific identification of genome-wide DNA-methylation differences between intracranial and extracranial melanoma metastases
Source: Sci Rep. 2023 Jan 9;13:444. doi: 10.1038/s41598-022-24940-w (PMC9829750; doi:10.1038/s41598-022-24940-w)
Supplement: Supplementary file 1 — Supplementary Information 1. [file 41598_2022_24940_MOESM1_ESM.pdf]

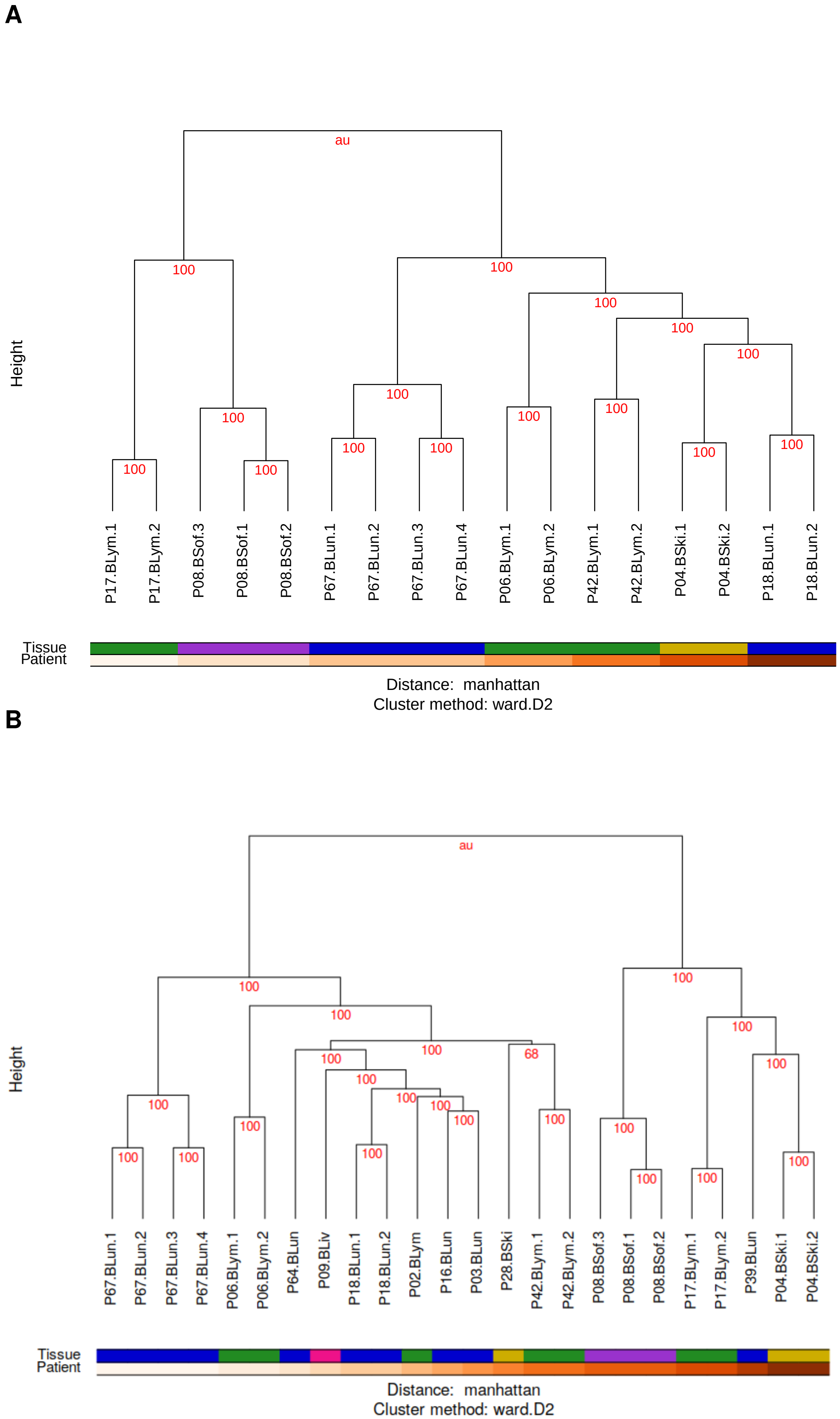

Figure S1: Hierarchical clustering of genome-wide DNA-methylation profiles of patient-specific metastases pairs to quantify similarities within the same and between patients. The red number above each subcluster in both dendrograms represents the approximate unbiased p-value (AU value) determined by bootstrapping, where a value of 100 means that the corresponding subcluster was completely stable. The tissue color coding below each dendrogram highlights the tissue type in which the extracranial metastasis of a patient-matched intra- vs. extracranial metastases pair occurred (green: lymph node, blue: lung, pink: liver, yellow: skin, purple: soft tissue). The patient-specific clustering of metastases pairs is further visualized by the patient color gradient below the dendrogram. **A**, Dendrogram obtained for the hierarchical clustering of all patient-matched pairs of patients that contained multiple samples of different histological regions of their metastases. All multiple pairs of each individual patient formed a completely stable subcluster. **B**, Dendrogram obtained for the hierarchical clustering of all patient-matched pairs also including those patients for which no multiple pairs were available. The observed stable clustering of all multiple pairs of individual patients (subpanel A) also remains completely stable when all patient-specific metastases pairs were considered.
