# Supplementary material for: Patient-specific identification of genome-wide DNA-methylation differences between intracranial and extracranial melanoma metastases
Source: Sci Rep. 2023 Jan 9;13:444. doi: 10.1038/s41598-022-24940-w (PMC9829750; doi:10.1038/s41598-022-24940-w)
Supplement: Supplementary file 2 — Supplementary Information 2. [file 41598_2022_24940_MOESM2_ESM.pdf]

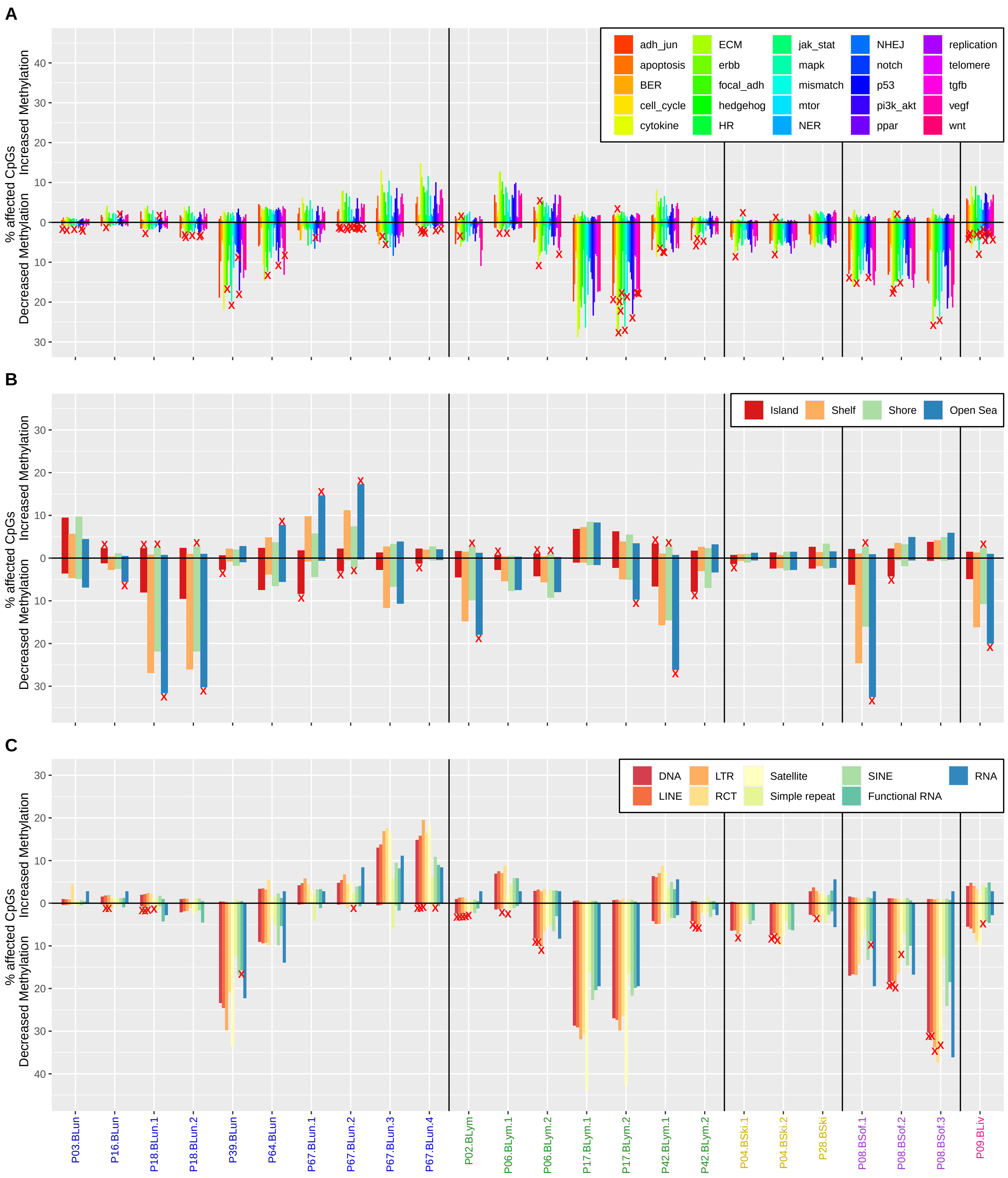

Figure S2: Patient-specific overview of differentially methylated CpGs predicted by the HMM-approach located in signaling pathway genes (A), CpG island categories (B), and transposon families (C). Significant enrichment of a category is marked with a red ’x’ (FDR-adjusted p-value < 0.05). The patient-specific metastases pairs are sorted by the tissue in which the extracranial metastasis occurred (x-axis) with brain vs. lung (blue), brain vs. lymph node (green), brain vs. skin (yellow), brain vs. soft tissue (purple) and brain vs. liver (pink).
