# Supplementary material for: Patient-specific identification of genome-wide DNA-methylation differences between intracranial and extracranial melanoma metastases
Source: Sci Rep. 2023 Jan 9;13:444. doi: 10.1038/s41598-022-24940-w (PMC9829750; doi:10.1038/s41598-022-24940-w)
Supplement: Supplementary file 3 — Supplementary Information 3. [file 41598_2022_24940_MOESM3_ESM.pdf]

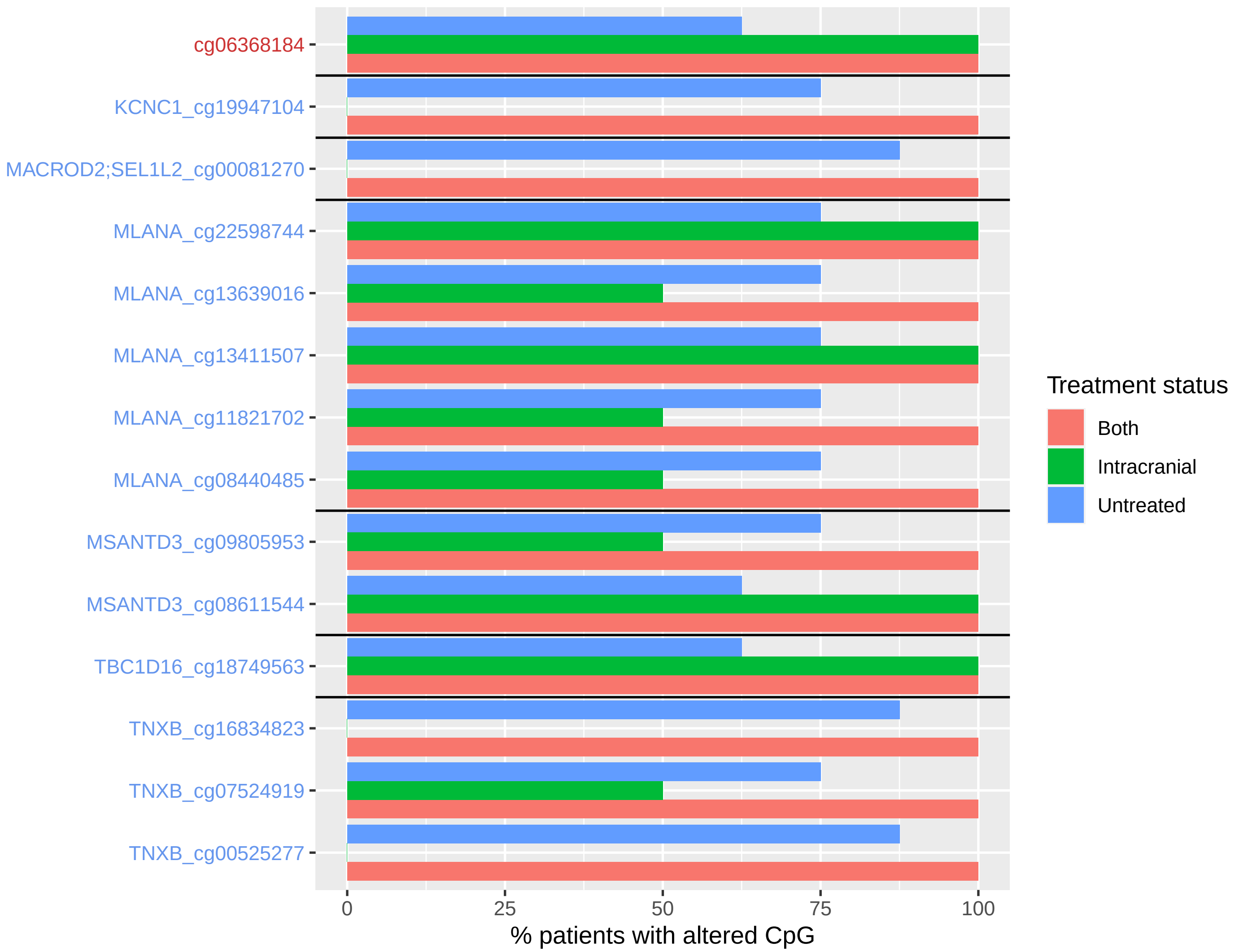

Figure S3: Analysis of the methylation states of the 14 top-ranking most discriminative CpGs of intra- and extracranial metastases in the context of the treatment status of patients. One CpG showed increased methylation in the intracranial metastases of at least 10 of 14 patients (cg06368184) and all other CpGs showed decreased methylation in at least 10 of 14 patients. The three individual bar plots for each CpG represent which percentage of patients with a specific treatment status had the corresponding methylation alteration. Black horizontal lines separate blocks of CpGs that were associated with specific genes. Associated gene names are shown in front of the CpG identifier along the y-axis. The treatment status is represented by three groups: both (red bars: patients with treatment against both metastases before surgical resection), intracranial (green bars: patients with treatment before resection of the intracranial metastasis), and untreated (blue bars: patients without treatment before the resection).
