# Supplementary material for: Patient-specific identification of genome-wide DNA-methylation differences between intracranial and extracranial melanoma metastases
Source: Sci Rep. 2023 Jan 9;13:444. doi: 10.1038/s41598-022-24940-w (PMC9829750; doi:10.1038/s41598-022-24940-w)
Supplement: Supplementary file 4 — Supplementary Information 4. [file 41598_2022_24940_MOESM4_ESM.pdf]

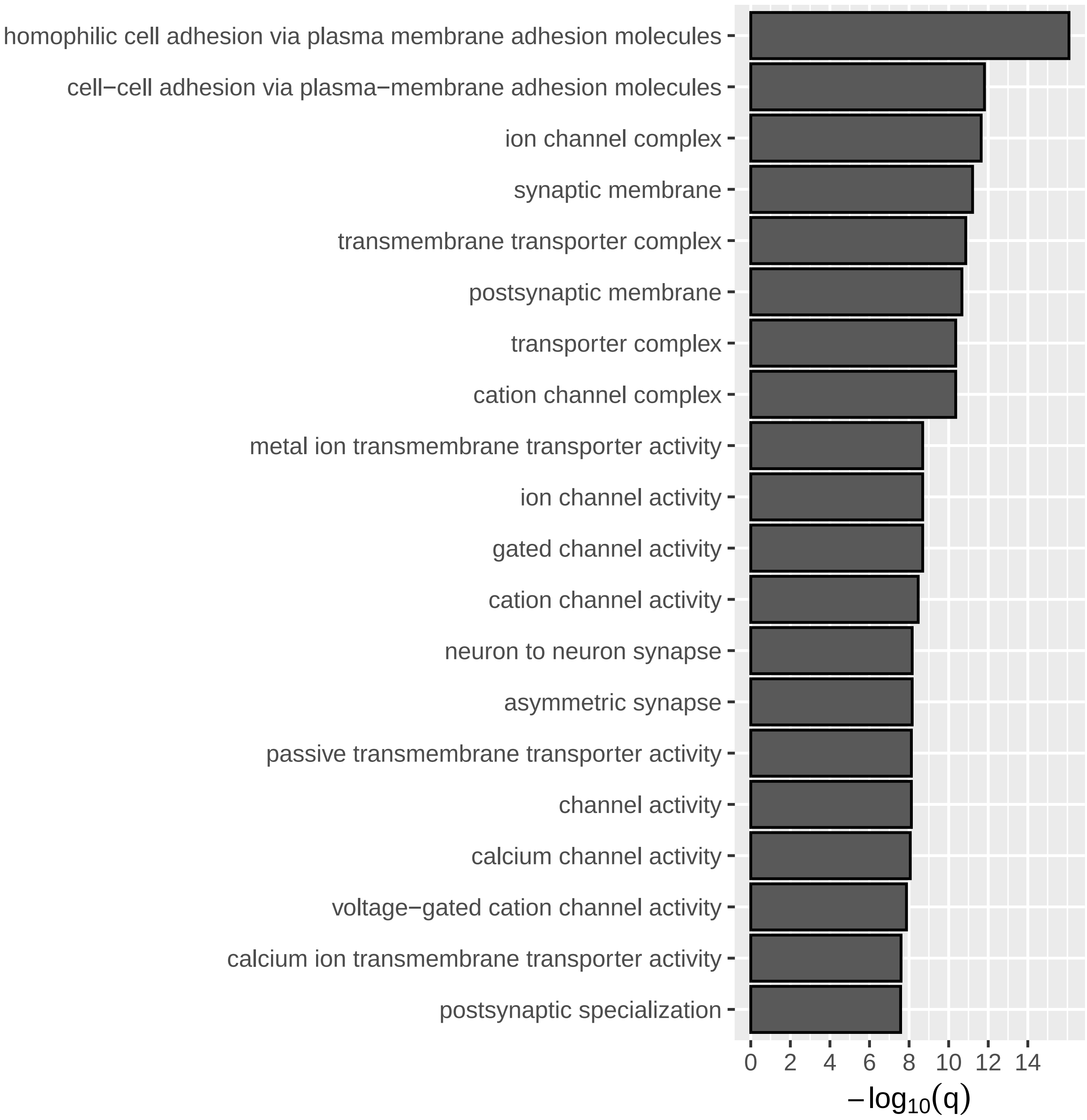


**Figure S4:** Enrichment of gene ontology (GO) terms for candidate genes that showed either decreased methylation (1,151 genes) or increased methylation (71 genes) of at least one CpG in at least 7 of 14 patients. The x-axis represents the -$\log_{10}$(q) of the obtained q-value and the y-axis shows the corresponding significantly enriched GO terms.
