# Supplementary material for: Patient-specific identification of genome-wide DNA-methylation differences between intracranial and extracranial melanoma metastases
Source: Sci Rep. 2023 Jan 9;13:444. doi: 10.1038/s41598-022-24940-w (PMC9829750; doi:10.1038/s41598-022-24940-w)
Supplement: Supplementary file 5 — Supplementary Information 5. [file 41598_2022_24940_MOESM5_ESM.pdf]

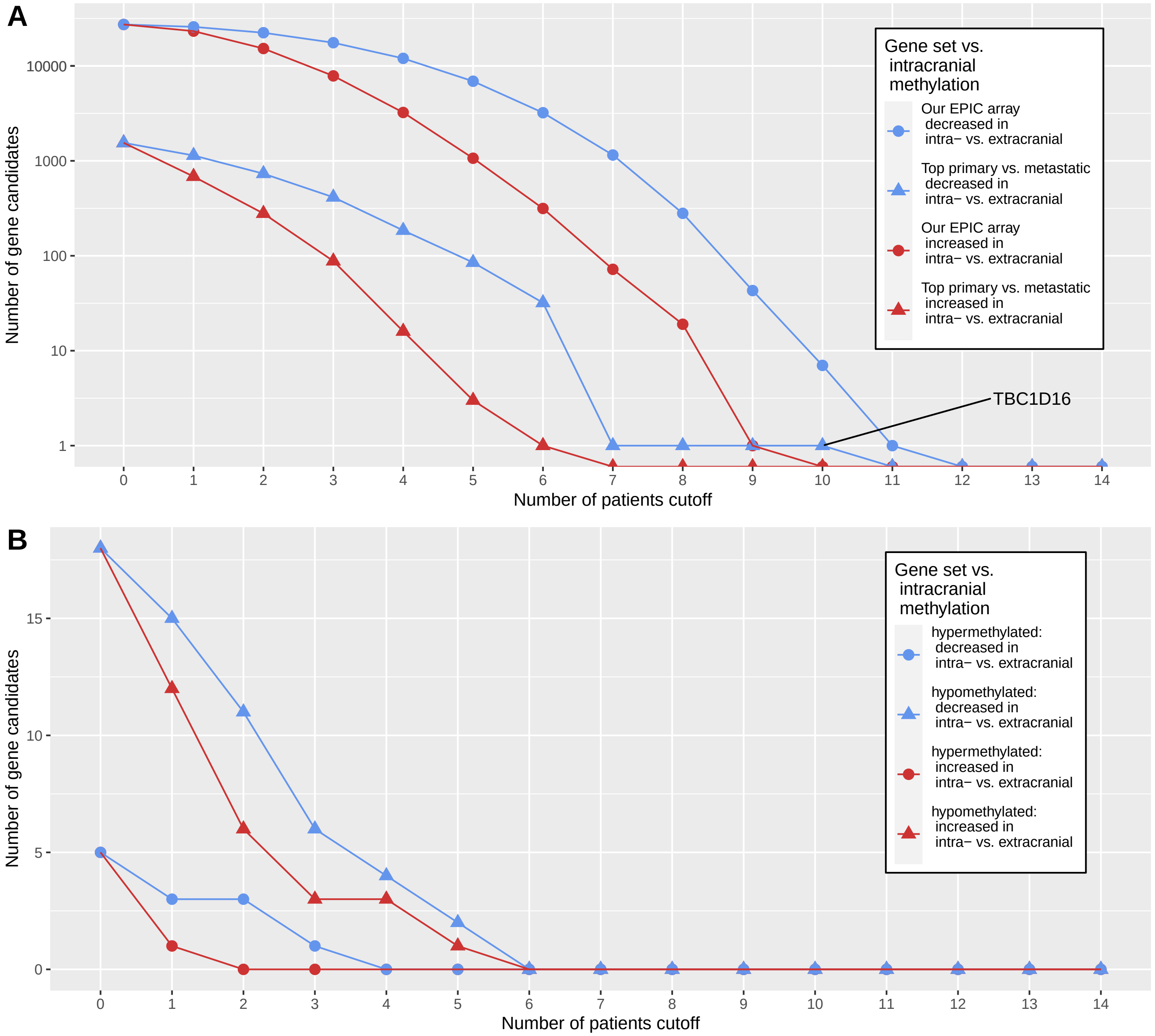

Figure S5: Number of gene candidates with decreased and increased methylation in intracranial metastases that were identified in at least a specific number of patients (x-axis) in relation to their corresponding extracranial metastases. **A,** Ranking of the 1,549 genes that were associated with the mappable 2,410 CpGs that were identified by Vizoso et al. (2015) to distinguish primary from metastatic melanoma cell lines. Methylation behavior of these genes in our patient cohort is represented by the two colored curves with the filled triangles showing how many genes were found to have decreased (blue) or increased methylation (red) in the intra- compared to the extracranial metastasis of our patient-specific metastases pairs. The top candidate gene *TBC1D16* that was found by Vizoso et al. (2015) to have lost its methylation in metastatic cell lines to trigger the metastatic cascade also showed a decreased methylation in the intra- compared to the extracranial metastasis for 10 of 14 of our patients. The two colored curves with the filled dots represent the methylation behavior of all genes in our study. **B,** Ranking of the 5 mappable genes covered by the 10 hypermethylated regions and of the 18 mappable genes covered by the 65 hypomethylated regions that have been identified by Chatterjee et al. (2017) to distinguish metastatic from primary matched melanoma cell lines. The colored curves represent the methylation behavior of the hypo- and hypermethylated gene set in the context of our patient cohort comparing patient-matched intra- and extracranial metastases.
