# Supplementary material for: Patient-specific identification of genome-wide DNA-methylation differences between intracranial and extracranial melanoma metastases
Source: Sci Rep. 2023 Jan 9;13:444. doi: 10.1038/s41598-022-24940-w (PMC9829750; doi:10.1038/s41598-022-24940-w)
Supplement: Supplementary file 6 — Supplementary Information 6. [file 41598_2022_24940_MOESM6_ESM.pdf]

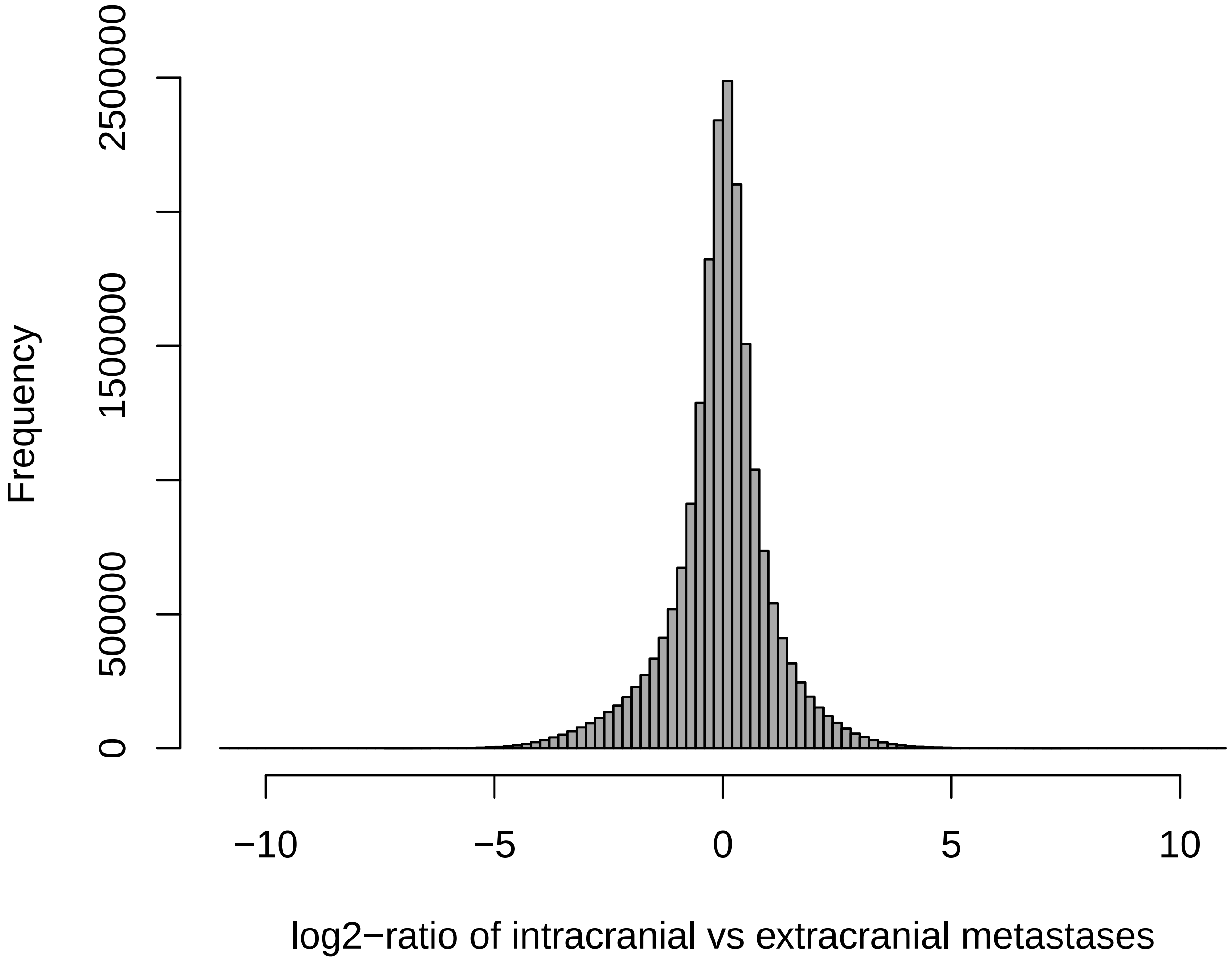

Figure S6: Histogram of CpG-specific $\log_2$-ratios comparing DNA-methylation measurements of intra- to extracranial metastases. Log_2-ratios of all patient-specific metastases pairs are included. Negative $\log_2$-ratios suggest decreased, $\log_2$-ratios about zero suggest unchanged, and positive $\log_2$-ratios suggest increased methylation of the underlying CpG in intra- compared to extracranial metastasis tissue.
